# Weight status and gender-related differences in motor skills and in child care - based physical activity in young children

**DOI:** 10.1186/1471-2431-12-23

**Published:** 2012-03-09

**Authors:** Antoine Bonvin, Jérôme Barral, Tanja H Kakebeeke, Susi Kriemler, Anouk Longchamp, Pedro Marques-Vidal, Jardena J Puder

**Affiliations:** 1 Institute of Sport Sciences, University of Lausanne, Bâtiments administratifs de Vidy, Route de Chavannes 33, 1015 Lausanne, Switzerland; 2 Child Development Center, University Children's Hospital, 8032 Zürich, Switzerland; 3 Swiss Tropical and Public Health Institute, University of Basel, Socinstrasse 57, 4002 Basel, Switzerland; 4 Health League, Rue de la Mouline 8, 1022 Chavannes-près-Renens, Switzerland; 5 Service of Endocrinology, Diabetes and Metabolism, Centre Hospitalier Universitaire Vaudois, University of Lausanne, Rue du Bugnon 46, 1011 Lausanne, Switzerland

**Keywords:** Children, Motor skills, Physical activity, Gender, Overweight, Youp'là bouge

## Abstract

**Background:**

Over the last decades, a decline in motor skills and in physical activity and an increase in obesity has been observed in children. However, there is a lack of data in young children. We tested if differences in motor skills and in physical activity according to weight or gender were already present in 2- to 4-year-old children.

**Methods:**

Fifty-eight child care centers in the French part of Switzerland were randomly selected for the Youp'là bouge study. Motor skills were assessed by an obstacle course including 5 motor skills, derived from the Zurich Neuromotor Assessment test. Physical activity was measured with accelerometers (GT1M, Actigraph, Florida, USA) using age-adapted cut-offs. Weight status was assessed using the International Obesity Task Force criteria (healthy weight vs overweight) for body mass index (BMI).

**Results:**

Of the 529 children (49% girls, 3.4 ± 0.6 years, BMI 16.2 ± 1.2 kg/m^2^), 13% were overweight. There were no significant weight status-related differences in the single skills of the obstacle course, but there was a trend (p = 0.059) for a lower performance of overweight children in the overall motor skills score. No significant weight status-related differences in child care-based physical activity were observed. No gender-related differences were found in the overall motor skills score, but boys performed better than girls in 2 of the 5 motor skills (p ≤ 0.04). Total physical activity as well as time spent in moderate-vigorous and in vigorous activity during child care were 12-25% higher and sedentary activity 5% lower in boys compared to girls (all p < 0.01).

**Conclusions:**

At this early age, there were no significant weight status- or gender-related differences in global motor skills. However, in accordance to data in older children, child care-based physical activity was higher in boys compared to girls. These results are important to consider when establishing physical activity recommendations or targeting health promotion interventions in young children.

## Background

The prevalence of overweight and obesity has reached epidemic levels [1], even in young children [2]. Moreover, fitness levels of school children have significantly declined over the three last decades [3], and a decline in some motor skills of preschoolers [4], as well as in physical activity of school children [5] has also been observed. In addition, physical activity and motor skills in children may underlie a reciprocal and dynamic relationship, which is mediated by factors such as aerobic fitness and obesity [6-8].

Weight status-related differences in motor skills [9,10] and in measured physical activity [11,12] are well documented in school children, though some controversy remains [13]. However, as prevention strategies are proposed to start at preschool age [14-18], it is also important to assess motor skills and physical activity levels in this younger age group in order to avoid large discrepancies. The few existing studies conducted in 4- to 6-year-old preschool children report controversial findings regarding weight status-related differences in motor skills [19-21] and in objectively measured physical activity [22,23]. To our knowledge, no studies investigating differences in motor skills and in physical activity between healthy weight and overweight children below age 4 have been performed.

In preschoolers, weight status-related differences in physical activity have been postulated to be gender-dependant with more pronounced differences in boys [23]. In this context, the question arises, if there are also gender differences in physical activity in preschoolers or in even younger children. Although previous studies hint to gender differences in infants (0 to 12 months) [24], data using accelerometers, the gold standard of physical activity measurements in a more epidemiological context [25], are lacking in children before entering preschool. In two studies [22,26], accelerometers have been used in 3- to 5-year-old preschoolers. In both studies, gender differences in physical activity were found with boys being more active than girls. However, this observation was not confirmed in another study [27]. Similarly, it remains controversial if gender differences in motor skills already exist in preschoolers [26,27]. Moreover, studies including children below age 4 are rather scarce.

Hence, in this study, we used data from the Youp'là Bouge study to investigate weight status and gender-related differences in motor skills and physical activity in 2- to 4-year-old children.

## Methods

**Figure 1:**
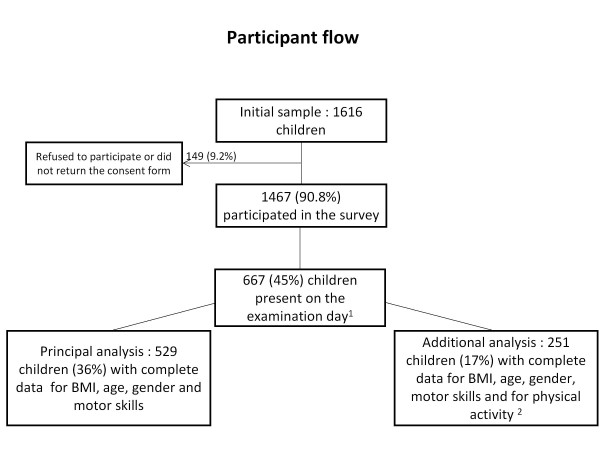
**Participant flow**. ^1^The numbers of children are due to the low attendance of children at their child care center (mean attendance 48 ± 2 6%). ^2^Physical activity measures were only performed in 30 out of 58 randomly selected child care centers.

The "Youp'là bouge" study (clinical trials.gov NCT00967460), is a randomized controlled trial conducted in 58 randomly selected public child care centers in urban and rural areas of the French-speaking part of Switzerland (cantons of Vaud, Neuchâtel and Jura). Thereby, the governmental institution that ran the program randomly selected 20 of the existing child care centers in each of the three cantons. Two centers withdrew before beginning the study. Thus, a total of 58 child care centers participated in the study. The present analysis focuses on the baseline data of this physical activity program. Half of the child care centers were then randomly assigned to the intervention (n = 29) or the control (n = 29) arm. The study was approved by the respective regional ethical committees (cantons of Vaud, Neuchâtel and Jura). After receiving the approval from all directors of the child care centers, written informed consent was obtained from the parents or legal representatives of 1467 of the 1616 initially selected children (participation rate: 91%, Figure 1). In each child care center, anthropometric and motor skills were simultaneously assessed on one randomly selected day of the week. As attendance of the children at their child care center was 48% ± 26% (mean ± standard deviation; 100% corresponding to 5 full weekdays from 9 am to 5 pm), 667 of the participating children were present on the selected examination days. Due to cost and logistic reasons, half of the child care centers of each arm (intervention and control) were randomly selected (total n = 30) to also include physical activity measurements which were performed one week after the other measures. The present analysis includes 529 children (36% of participating children) who had valid data for body mass index (BMI), age, gender and all 5 motor skills of the obstacle courses. There were no differences between children with and without a valid dataset regarding gender, but the former were slightly older (3.4 ± 0.6 and 3.2 ± 0.7, p < 0.001).

### Anthropometry

Standing height was determined and body weight was measured using an electronic scale (Seca, Basel, Switzerland; accuracy 0.05 g). BMI was calculated as weight kg/m^2^. Children were classified into two BMI-groups "healthy weight" and "overweight" group (including both overweight and obese children) according to the International Obesity Task Force criteria [28].

### Motor skills

**Figure 2:**
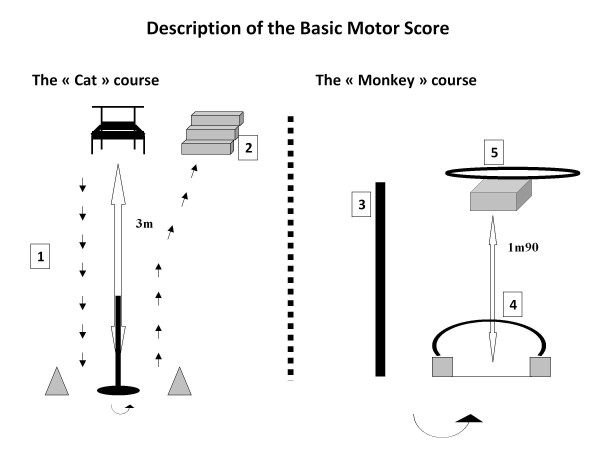
**Description of the Basic Motor Score**. ^1^Running, ^2^Climbing up and down the stairs, ^3^Balancing, ^4^Getting up, ^5^Landing after jumping.

Motor skills measures were adapted from the Zurich Neuromotor Assessment (ZNA) test, a standardized and reliable test for 5- to 8-year-old children [29,30]. This adaptation and an extension of the test for 3- to 5-year-old children has been recently developed [31] (Figure 2). This test was developed for this age group based on the developmental stages (initial, elementary and mature) according to Gallahue and al. [32]. In a pilot study conducted in two additional child care centers, we computed the test-retest reliability over a two weeks time period (n = 33, r = 0.5, p < 0.05 for both evaluators) and the inter-rater correlation (n = 42, r = 0.7, p < 0.05) of the total Basic Motor Score (sum of the 5 motor skills, see below) for two of the three evaluators of the present study.

In this test, the 5 motor skills were performed in two playful obstacle courses (the Cat and the Monkey) and each task was rated on a 5-point scale scoring from 0 for worst to 4 for best. Motor skills testing were carried out in a separate room in groups of 4 to 6 children in the presence of 1 educator and 3 evaluators. Each of both obstacle courses was explained and demonstrated to a subgroup of 2 to 3 children and each child was evaluated and scored individually by one evaluator. This test allows assessing motor skills performance of 6 children with two evaluators within 15 minutes.

In the "Cat" course (Figure 2), children stood up from a chair, ran 3 meters to a pole, turned around it, ran back and climbed up and down a three-step stairs while removing a sticker from the wall on the top of the stairs. The motor skills running (1) and climbing up and down the stairs (2) were scored.

In the "Monkey" course (Figure 2), children balanced on a beam, passed under a tunnel, got up and jumped from a case (height of the first step of the stairs). The motor skills balancing (3), getting up (4) and landing after jumping (5) were scored. To further differentiate children with excellent motor skills, children were additionally asked to jump on one leg as many times as they could. Each motor skill was evaluated using a scale ranging from 0 (unable to perform the task) to 4 (excellent). Refusals (n = 60 out of 589) were removed from the analyses. Two motor scores were calculated to determine an overall motor skills score: 1) an "Integral Motor Score" (sum of 6 motor skills) that included the additional and most difficult task "jumping on one leg". This score ranged from 0 (unable to perform all 6 motor skills) to 24 (best performance on all tasks) and 2) a "Basic Motor Score" (sum of 5 motor skills, regarded as the reference score) that did not include this task and whose score ranged from 0- 20. Children, who refused any of the tasks, could not be included in these motor skills score.

### Child care-based physical activity

Physical activity was measured over only one day at the child care center with an accelerometer (GT1M, Actigraph, Florida, USA). The accelerometer was worn around the hip and programmed to save data in 15 s intervals (epoch size of 15 s), as proposed for this age [25,33]. The CSA/Actigraph is the most commonly used motion sensor in children and has a good reproducibility, validity and feasibility [34]. This physical activity assessment has been shown to be valid across different activities in 3 to 5 years old children with a Pearson correlation coefficient between VO_2 _(ml/kg per min) and Actigraph counts/epoch of *r *= 0.82 [25]. Data was considered as valid if collected for at least 3 hours. This allowed the inclusion of children attending the child care center during half days. Mean total wearing time was 6.1 ± 1.4 hours. Sequences of at least 10 min of consecutive zero values were removed and interpreted as accelerometer not worn [35]. Child care-based total physical activity level was expressed in counts per minute (cpm, total counts recorded divided by total daily wearing time). Physical activity was further categorized into moderate-vigorous physical activity (MVPA), vigorous physical activity (VPA) and sedentary activity. The following age-adjusted cut points were used to define MVPA, VPA and sedentary activity [25]: ≥ 420 counts/epoch for MVPA; ≥ 842 counts/epoch for VPA and < 37.5 counts/epoch for sedentary activity. Data are expressed as the number of epochs/hour above the respective cut-offs. Valid physical activity data were obtained for 275 children, as only half of the child care centers were selected for physical activity measurements. To be able to investigate the same population, physical activity data were only used for those 251 children who also had valid Basic Motor Score measures (Figure 1).

### Statistics

Statistical analyses were performed using STATA version 11.0 (Statacorp, College Station, Tx, USA). Descriptive results were presented as mean ± standard deviation (SD) for quantitative variables and as percentages for qualitative variables. Differences in age and gender between children with and without a complete dataset were calculated using mixed linear or logistic regression models with child care center (= cluster) as random effect. The differences in anthropometry, motor skills and physical or sedentary activity according to weight status or gender were also calculated using mixed linear or logistic regression models with child care center as random effect. These models were also adjusted for age and, weight status-related differences also for gender. Differences in motor skills were further adjusted for total physical activity (counts/minutes). Between-gender differences in physical activity were also expressed in percentages using girls' physical activities as a denominator. We tested weight status by gender, weight status by age or gender by age interactions. We also tested, if BMI was related to motor skills or activity measures after adjustment for age and gender. Statistical significance was assumed at *p *< 0.05.

## Results

### Characteristics of the participating children

Table 1 shows the characteristics of the 529 children with a valid dataset (i.e. who had complete data for BMI, age, gender and for all 5 motor skills of the Basic Motor Score). There were no significant gender differences in the anthropometric measures, although overweight prevalence tended to be higher in girls (p = 0.053).

**Table 1:** Children's characteristics

	**Total sample**	**Boys**	**Girls**	**P-value**
	
n (%)	529	268 (51%)	261 (49%)	
Age (years)	3.4 ± 0.6	3.4 ± 0.6	3.4 ± 0.6	0.9
Weight (kg)	15.8 ± 2.2	15.9 ± 2.0	15.6 ± 2.3	0.08
Height (cm)	98.5 ± 6.2	98.8 ± 5.7	98.1 ± 6.7	0.1
Body mass index	16.2 ± 1.2	16.3 ± 1.2	16.2 ± 1.3	0.3
(kg/m^2^)				
Overweight	12.9	10.1	15.7	0.06
children (%)^a^				

Values are means (± standard deviations), unless stated differently.

^a ^according to the International Obesity Task Force percentiles

No weight status-related differences were found in the single motor skills (p > 0.1). However, a tendency for lower performance in the overweight children was observed in the total Basic Motor Score (n = 529, p = 0.059), but not in the total Integral Motor Score (n = 411, p = 0.19) (Table 2). However, the differences in the total Basic Motor Score did not remain significant after having limited the sample size to only those children who also had a valid Integral Motor Score (n = 411, p = 0.13). No significant differences were found in total physical activity including sedentary activity between healthy weight and overweight children (Table 2, all p ≥ 0.6). We also tested if BMI was related to motor skills or activity measures: after adjustments for age and gender, increased BMI was related to a decreased score in the running task (beta coefficient of -0.14 and 95% CI of -0.28 to -0.08, p < 0.05), but not to the other single motor skills (p > 0.06), both Motor Scores (both p > 0.25) or any of the activity measures (all p > 0.2).

**Table 2:** Motor skills, physical activity and sedentary activities according to weight status

		Weight Status	
	**Healthy Weight**	**Overweight **^b^	**ß-coefficient (95% CI)**
	
**Motor Scores **^a^	***n = 461***	***n = 68***	
*Basic Motor Score*	12.59 ± 3.43	11.91 ± 3.55	-0.65 (-1.33 to 0.02)
	***n = 356***	***n = 55***	
*Integral Motor Score*	14.41 ± 3.82	13.43 ± 3.81	-0.57 (-1.42 to 0.28)
	
**Physical Activity**	***n = 214***	***n = 37***	
Total physical activity (counts/min)	610 ± 211	587 ± 201	-11.8 (-75.4 to 51.8)
Sedentary activity (epochs/hour < 37.5 counts)	130 ± 22	132 ± 24	1.6 (-5.4 to 8.5)
MVPA (epochs/hour ≥ 420 counts)	29 ± 13	29 ± 13	1.1 (-3.0 to 5.1)
VPA (epochs/hour ≥ 842 counts	8 ± 6	7 ± 5	-0.1 (-1.8 to 1.6)

Values are means ± standard deviation and β-coefficients with 95% confidence intervals.

Variables were analyzed using a multiple linear regression, adjusted for age, gender and child care center (cluster).

^a ^Basic Motor Score: Reference score for motor skills: sum of the 5 motor skills (running, climbing stairs, balancing on a beam, getting up, landing after jumping); Integral Motor Score: sum of 6 motor skills including the jumping on one leg motor skill

^b ^according to the International Obesity Task Force percentiles Significant gender differences (* *p *< 0.05, **p < 0.01, ***p < 0.001).

MVPA: moderate-and-vigorous physical activity; VPA: vigorous physical activity

Boys performed better than girls in running and climbing stairs (p = 0.039 and p = 0.003, respectively). However, no significant differences were found in the other motor skills, the Basic Motor Score or the Integral Motor Score (Table 3, all p ≥ 0.3). Conversely, boys had significantly higher total physical activity, and spent more time in MVPA and VPA and less time in sedentary activity than girls (Table 3, all p ≤0.01). This corresponds to a difference in total physical activity of 12%, in MVPA of 22%, in VPA of 25% and in sedentary activity of 5%. Differences in motor skills running and climbing stairs were no longer significant after adjustment for total physical activity (p = 0.09 and p = 0.21, respectively).

**Table 3:** Motor skills, physical activity and sedentary activities according to gender

		Gender	
**Motor Scores **^a^	**Boys**	**Girls**	**ß-coefficient (95% CI)**
	
*Basic Motor Score*	***n = 268***	***n = 261***	
	12.56 ± 3.62	12.44 ± 3.27	-0.18 (-0.64 to 0.27)
	***n = 210***	***n = 201***	
*Integral Motor Score*	14.27 ± 4.06	14.28 ± 3.58	-0.27 (-0.85 to 0.30)
	
**Physical Activity**	***n = 120***	***n = 131***	
Total physical activity (counts/min)	641 ± 220	574 ± 195	-68.7 (-115.0 to -22.3) **
Sedentary (epochs/hour < 37.5 counts)	127 ± 23	134 ± 20	6.5 (1.4 to 11.5) **
MVPA (epochs/hour ≥ 420 counts)	32 ± 14	26 ± 11	-6.2 (-9.1 to -3.3) ***
VPA (epochs/hour ≥ 842 counts)	9 ± 6	7 ± 5	-1.8 (-3.0 to -0.5) **

Values are means ± standard deviation and β-coefficients with 95% confidence intervals. Variables were analyzed using a multiple linear regression, adjusted for age and child care center (cluster).

^a ^Basic Motor Score: Reference score for motor skills: sum of all 5 motor skills (running, climbing stairs, balancing on a beam, getting up, landing after jumping); Integral Motor Score: sum of 6 motor skills including the jumping on one leg skill Significant gender differences (*p < 0.05, **p < 0.01, ***p < 0.001).

MVPA: moderate-and-vigorous physical activity; VPA: vigorous physical activity.

Restricting all analyses related to motor skills to the children with valid physical activity measures (n = 251) did not change the results regarding motor skills, except that overweight children performed less well in the balancing on a beam skill (p = 0.046). Gender differences remained significant in running (p = 0.004), but not in climbing stairs (p = 0.11).

Finally, no significant weight status by gender, weight status by age or gender by age interactions were observed for motor skills and physical activity (all p > 0.08).

## Discussion

The goal of our study was to evaluate differences in motor skills and in physical activity according to weight or gender in 2- to 4-year-old children attending child care center. No significant weight status- or gender-related differences in overall motor skills were found. However, total physical activity and time spent in MVPA and VPA were higher while sedentary activity was lower in the 2- to 4-year-old boys compared to girls.

To our knowledge, weight status-related differences have not been studied in children that young. A few studies in 4- to 6-year-old preschoolers examined differences in motor skills according to weight status [19-21,36], and their results were controversial. One study found no differences [36], while several other studies [19-21] found globally better motor skills (particularly in the more dynamic tests) in healthy weight compared to overweight children. The reasons for the discordance between studies are not clear as many of the tests applied (locomotion, agility and balance) were quite similar. The authors hypothesized that the differences might be due to the fact that performance differences may become apparent at a later age and subsequently increase with age [21,36]. We would need more studies to investigate this hypothesis, as it is refuted by a study in school children that did not find any weight-related differences in motor skills [13]. However, our data, generated in a relatively large population of over 500 2-to 4-year-old children, would be in accordance with this assumption: We found a trend for a lower motor performance in overweight children, but no significant weight status-related differences in motor skills. Yet, the few number of overweight children might have reduced our power. Using weight status as a categorical variable is a crude and conservative way to test for the effect of weight on motor performance. However, using BMI instead of weight status did not change the results in a relevant way. The lack of large significant differences in motor skills in this age group highlights the importance of an early prevention to reduce the barriers of later overweight related to physical fitness. Thereby, the child care center may represent an ideal setting.

No weight status-related differences in total physical activity, MVPA, VPA or sedentary activity were found. In school children, differences in physical activity between healthy weight and overweight children are well documented [11,12]. Even in a defined setting during school time, 8- to 10-year-old healthy weight children spent more time in MVPA compared to overweight children [11]. Data in preschool children are more controversial [22,23]. In a small study (n = 56), Metallinos and al. [22] observed that overweight 3- to 5-year-old children spent significantly less time in VPA than their healthy weight counterparts, but total physical activity (counts per minute) did not differ. In another study investigating 3-to 5-year-old children [23], significant weight status-related differences in time spent in MVPA and VPA were found in boys (n = 118), but not in girls (n = 127). Again, total physical activity did not differ. Niederer et al. [21] studied 4- to 6-year-old children (n = 613) and found no differences in total and VPA between healthy weight and overweight children, but significant differences in these measures between healthy weight and obese children. Differences between studies could be explained by age or methodological differences such as cut-offs to define overweight/obesity [28,37,38] or the different physical activity intensities [25,39,40]. The relatively low PA cut-offs compared to cut-offs [41] used in other studies may also have contributed to the absence of significant differences. Previous reports [21-23] did not compare sedentary activity between healthy weight and overweight preschoolers, although sedentary activity is an important measure in regards to overweight and cardiovascular risk [42]. In the current study, no differences were found between healthy weight and overweight children regarding sedentary activity. Overall, our results and the results of previous studies suggest that weight status-related differences in motor skills and in physical activity start to develop around the preschool age and these data underline the importance of early preventive activities.

In the current study, boys performed better than girls in two motor skills (running and climbing stairs), but these differences were no longer significant after summing up the motor skills into the Basic Motor Score. The difference in these two motor skills did also not remain significant after adjusting for differences in physical activity. As we noticed a gender difference in physical activity, these results might imply that girls' motor skills could have improved if they were getting the same amount of physical activity as boys. Interestingly, a study in preschoolers [43] observed that girls in the highest motor skill tertile spent significantly less time in VPA than boys in the highest tertile. Thus, the relationship between PA and motor skills seems to be complex and may also depend on the intensity of physical activity and the level of motor skill performance. Our results regarding motor performance are to some extent in accordance with the literature. Although Cliff and al. [27] observed in a small study (n = 46, 4.3 ± 0.7 years) that girls performed better than boys, Fisher and al. [26] found no gender differences in a study including 394 children (age 4.2 ± 0.5 years). Differences in sample size and the choice of motor tasks might explain these contradictory findings. Although the motor test used by Fisher and al. [26] (Movement Assessment Battery) [44] is quite similar to the one used by Cliff and al. [27] (Test of Gross Motor Development) [45], some skills were evaluated in one study but not in the other (balance and skips in the Fisher and al. [26]; run, gallop, leap, slide and ball dribble in the Cliff and al. [27]). Taking the two larger studies together (Fisher et al. and the current one), there seems to be no significant gender differences in global gross motor skills in young children. However, it would be interesting to investigate different gross motor skills domains (i.e. locomotive, manipulative or balancing tasks) in order to explore more precisely potential gender differences, as suggested previously [26].

Contrary to motor skills, we found significant gender differences in total physical activity as well as in the time spent in MVPA, VPA and in sedentary activity. Interestingly, larger differences were observed in the more intense physical activities. Although we only measured over a 8 hour period during one child care day (mean wearing time of 6 hours), our results show that even in a defined setting like child care center, with the same space and a globally similar timetable, boys are more active and less sedentary than girls. This finding is in accordance with previous studies in older children [22,26]. Similarly, data in infants, albeit not with accelerometry, confirm our data [24]. Our observed gender differences were not modulated by age and thus were equally present in 2- to 3-year-old children compared to their 4 year-old counterparts. As differences are observed at any age throughout lifespan [22,24,26,46,47], one wonders if gender differences in physical activity are innate or culturally-driven (i.e. impact of "nature versus nurture"). Regardless, child care center educators should be sensitized to these found differences in order to minimize gender inequities in physical activity opportunities in young children.

The present study has a number of limitations. The child care-based investigation of physical activity was performed during one single day (mean wearing time of 6 hours) and only during child care which may not be representative for the whole day, or be representative of a child care day. Considering the literature a minimum of three days would have been more valuable for this assessment [48]. However, Trost and al. suggest that the variability of this assessment observed over a single day seems to decrease as children get younger. Measurement restricted to the child care center setting can also be an advantage allowing detecting differences in a defined setting. But we cannot differentiate whether the social and physical environment in the respective child care center was more attractive to boys than for girls or whether boys may be genetically more active than girls. The motor skills assessment for this specific age has been validated [31]. In our own pilot study, test-retest and inter-rater correlations were 0.5 and 0.7, respectively. Compared to other motor tests [43-45], reliability and validity of the motor skills tests are only moderate. However, this test has the advantage to assess motor skills performance in a relatively short time, as six children can be assessed within 15 minutes if two raters participate.

Strengths of the study are its large sample size, the investigation of motor skills and of child care-based objectively measured physical activity and the young age of the children.

## Conclusions

In conclusion, our study provides novel information about weight status and gender-related differences in a large sample of 2- to 4-year-old children attending child care center. At this young age, overall motor skills and physical activity do not significantly differ according to weight status. Though there were no gender differences in overall motor skills, boys had higher physical activity level and were less sedentary than girls in this defined setting. These results are important to consider when establishing physical activity recommendations or targeting health promotion interventions in young children.

## Competing interests

The authors declare that they have no competing interests.

## Authors' contributions

JJP designed the study and is the principal investigator. JJP, JB and SK established the methods and questionnaires. TK assisted with the motor skills testing and SK with the accelerometry. AB coordinated and conducted the study with the help of JB, AL and JJP. PM gave statistical support. AB wrote the article with the support of JJP. JJP obtained the funding. All authors provided comments on the drafts and have read and approved the final version.

## Pre-publication history

The pre-publication history for this paper can be accessed here:

http://www.biomedcentral.com/1471-2431/12/23/prepub
